# Cost comparison of a rapid results initiative against standard clinic-based model to scale-up voluntary medical male circumcision in Kenya

**DOI:** 10.1371/journal.pgph.0000817

**Published:** 2023-03-29

**Authors:** Katrin Jaradeh, Tyler Van Fleet Kingery, Jackline Cheruiyot, Francesca Odhiambo, Elizabeth A. Bukusi, Craig R. Cohen, Starley B. Shade

**Affiliations:** 1 School of Medicine, University of California, San Francisco, San Francisco, California, United States of America; 2 Department of Epidemiology and Biostatistics, University of California, San Francisco, San Francisco, California, United States of America; 3 Centre for Microbiology Research, Kenya Medical Research Institute, Nyakach, Kenya; 4 Centre for Microbiology Research, Kenya Medical Research Institute, Kisumu, Kenya; 5 Department of Obstetrics, Gynecology and Reproductive Sciences, University of California, San Francisco, San Francisco, California, United States of America; University of Ghana, GHANA

## Abstract

Voluntary male medical circumcision (VMMC) reduces HIV acquisition by up to 60%. Kenya has successfully scaled up VMMC to an estimated 91% of eligible men and boys in certain regions in combination due to VMMC and cultural circumcisions. VMMC as a program is implemented regionally in traditionally non-circumcising counties where the prevalence is still below 91%, ranging from 56.4% to 66.7%. Given that funding toward VMMC is expected to decline in the coming years, it is important to identify what models of service delivery are most appropriate and efficient to sustainably meet the VMMC needs of new cohorts’ eligible men. To this end, we compared the costs of facility-based VMMC and one within a rapid results initiative (RRI), a public health service scheduled during school holidays to perform many procedures over a short period. We employed activity-based micro-costing to estimate the costs, from the implementer perspective, of facility-based VMMC and RRI-based VMMC conducted between October 2017 and September 2018 at 41 sites in Kisumu County, Kenya supported by the Family AIDS care & Education Services (FACES). We conducted site visits and reviewed financial ledger and programmatic data to identify and quantify resources consumed and the number of VMMC procedures performed during routine care and RRIs. Ledger data were used to estimate fixed costs, recurring costs, and cost per circumcision (CPC) in United States dollar (USD). A sensitivity analysis was done to estimate CPC where we allocated 6 months of the ledger to facility-based and 6 months to RRI. Overall, FACES spent $3,092,891 toward VMMC services and performed 42,139 procedures during the funding year. This included $2,644,910 in stable programmatic costs, $139,786 procedure costs, and $308,195 for RRI-specific activities. Over the year, 49% (n = 20,625) of procedures were performed as part of routine care and 51% (n = 21,514) were performed during the RRIs. Procedures conducted during facility-based cost $99.35 per circumcision, those conducted during the RRIs cost $48.51 per circumcision, and according to our sensitivity analysis, CPC for facility-based ranges from $99.35 to $287.24 and for RRI costs ranged from $29.81 to $48.51. The cost of VMMC during the RRI was substantially lower than unit costs reported in previous costing studies. We conclude that circumcision campaigns, such as the RRI, offer an efficient and sustainable approach to VMMC.

## Introduction

Voluntary medical male circumcision (VMMC) reduces female-to-male HIV transmission by 50–60% [1–3]. Prior to the roll-out of VMMC across Africa, the prevalence of male circumcision varied substantially among and within countries nationally and regionally due to religious, cultural and social practices [4, 5]. For example, the majority of communities in Kenya traditionally practiced male circumcision, nationally before VMMC roll out, 85% of men had been circumcised as of 2007; however, regionally in the former Nyanza Province which is predominantly inhabited by the Luo, a traditionally non circumcising community, only 48% of men had been circumcised [6–8]. VMMC as a program is implemented regionally in traditionally non-circumcising counties where the prevalence is still low.

In 2007, the World Health Organization (WHO) and United National Agency HIV/AIDS (UNAIDS) set the goal to achieve 80% of males 15–49 years old circumcised by 2020 in 14 priority countries in southern and eastern Africa [9]. Between 2008 to 2019, approximately 26.8 million total male circumcisions were performed in the WHO designated priority countries. Of those, 8% (more than 2 million) VMMC were conducted in Kenya [10].

In 2016, modeling data from Malawi suggested that scaling up VMMC services to those younger than 15 years of age is an investment for HIV prevention in the future [11]. In response to these data, countries have begun to focus VMMC on males aged 10–29 [12]. In 2007, the World Health Organization (WHO) and the Joint United Nations Programme on HIV/AIDS (UNAIDS) identified 15 priority countries that had low levels of male circumcision and high prevalence of HIV in Southern and Eastern Africa (Botswana, Eswatini, Ethiopia, Kenya, Lesotho, Malawi, Mozambique, Namibia, Rwanda, South Africa, South Sudan, Tanzania, Uganda, Zambia, and Zimbabwe) [12]. As of 2021, data from these 15 target countries in Africa indicates that 90% of males between the ages of 10–29 have received VMMC [11]. However, regional variation in the prevalence of circumcision remains. For example, as of 2018 in Kisumu County, Kenya (located in the previous Nyanza Province), 45% of males remain uncircumcised amongst men 15–64 years old [13].

As countries switch from donor to nationally supported programs, cost-efficient services will help ensure long-term sustainability of VMMC [14]. Innovative and sustainable strategies are needed to reach remaining young men and to continue offering circumcision for new cohorts of boys as they come of age. Different models of VMMC delivery exist which include permanent sites within a large healthcare facility, deployment of health care providers to smaller health facilities, and mobile services that send health care personnel to non-health care facilities such as schools, churches, and places of work [15]. Various governmental and non-governmental institutions have stepped in and implemented Rapid Results Initiatives (RRI) in tandem with facility-based circumcisions to increase access and reach a large number of males over a short period of time. Traditionally, RRIs are high output campaigns that help mobilize resources and offer boys and men VMMC during school holidays [16].

However, few studies have estimated the costs of different models of VMMC service delivery. In 2012 a study reported VMMC costs around $66 per procedure in Kenya when using multi-site facility sampling [17]. Shortly after, in 2015, a study conducted in Uganda noted that cost per VMMC in a mobile site was between $60.79-$72.21 [18]. In 2016 and 2018 the cost of one procedure was $108 during an education-based VMMC intervention for street-connected youth in Kenya (30% which covered personnel costs) [19, 20]. However, few studies have compared the efficiency of facility-based and RRI-based VMMC among men of all ages [21].

In response to the scarcity of data on the costs of different models of VMMC implementation, we conducted a study to compare the costs per circumcision (CPC) within routine facility-based care and RRI within Kisumu County, Kenya. This study was conducted in collaboration with the Kenya Ministry of Health, Kenya Medical Research Institute, and Family AIDS Care & Education Services, a Center for Disease Control/President’s Emergency Plan for AIDS Relief (CDC/PEPFAR) implementing partner.

## Materials and methods

### Program description

Facility-based VMMC are conducted at a stand-alone medical clinic where a patient can present for a circumcision anytime of the year. These locations typically have personnel and supplies onsite year-round. Teams conducting these procedures consist of a surgically trained clinical officer, a surgical assistant, an infection control officer, an HIV testing counselor, and outreach worker. Resources include surgical rooms, recovery rooms, surgical supplies, and autoclaves. Activities include infection testing capabilities, surgery, surgical 1 week follow-up, and infection prevention counseling.

In 2009, the Kenya National AIDS and STI’s Control Programme (NASCOP) and the Ministry of Health (MOH) introduced RRIs as part of the campaign to increase the number of VMMC conducted throughout Kenya. RRIs typically take place during school holidays to target school-aged boys. During these school holiday campaigns, multidisciplinary teams are assembled, and programs enlist permanent surgical team staff, per diem surgical team staff, and social mobilization personnel to raise awareness about the RRI campaign [22]. During an RRI: staff carry out intense public outreach two weeks prior to the campaign to increase awareness about the RRI campaign; prepackaged kits are distributed to RRI teams; and to ensure an early start to the RRI, some participants meet with their surgeon prior to the campaign [22]. During an RRI there are various cost differences from facility-based VMMC. These include per-diem surgical staff, transportation for staff and patients, and in some settings, site rental expenses. RRIs included in our costing activity were implemented during school holidays which occurred in December 2017, April 2018, and August 2018.

### Study setting

We employed an activity-based micro-costing approach to estimate costs of VMMC conducted within routine care (facility-based VMMC) and within RRIs between October 2017 and September 2018 across Kisumu County, Kenya. We assessed costs associated with VMMC across 41 sites which are supported by FACES in Kisumu County [22]. Ethical approval was provided by the institutional review boards at Kenya Medical Research Institute in Kenya and at University of California San Francisco in the United States. This project was also reviewed and approved in accordance with the US CDC human research protection procedures and was given a non-research determination. Informed consent was not obtained as investigators did not interact with human subjects and had no access to identifiable data or specimens for research purposes.

### Data collection

We conducted in-person site visits to define resources used for facility-based VMMC and RRI campaigns. In each site, we conducted informal interviews with in-charge clinicians and VMMC staff to assess the resources used in the facility and during an RRI to support VMMC. The team appraised the supplies used such as anesthesia, surgical tools, cleaning/autoclave, personnel needed for the site to function, per diem staff, travel costs, and utilities.

### Programmatic data

FACES staff collected data from each clinic every month on the number of medical circumcisions conducted by age group as part of routine program reporting. Reporting include categorization of VMMC into 3 different age groups (10–14 years old, 15–24 years old, and 25+ years old).

### Ledger data

We reviewed ledger data from the FACES program, payroll from the Kenya Medical Research Institute (KEMRI), and payroll from the Kenya Ministry of Health (MOH) for the duration of our study period from 2017–2018 to identify money allocated for salaries. The exchange rate for the study period (2017–2018) was used to report on spending values from Kenyan shilling (KSH) to United States Dollar (USD).

### Data categorization

Using the ledger data, we categorized each line item into standard costing categories: 1) Recurrent Goods, which include items that require recurrent purchases and payments; 2) Capital Goods, which include large purchases for equipment used over many years; 3) Facility costs, which include rent of locations where VMMCs were performed; 4) Personnel, which includes MOH, KEMRI and per diem employees who participate in mobilization, HIV testing, surgical procedures, follow-up, transportation, or sanitation; and 5) Services, which include transportation, printing and reproduction (S1 Table).

To accurately represent costs per procedure in our intervention, we further categorized all spending into broad categories with a total of 49 different groups including: insurance, office supplies, rent, utilities, value added tax (VAT), facilities, per-diems, transportation, etc. S2 Table. Next, we assigned all 49 groups of spending into the following groups: 1) stable programmatic costs (example: personnel and benefits, utilities, etc.), 2) RRI-specific costs (example: per diem staff, etc.), or 3) per procedure costs (example: surgical supplies, participant incentives, etc.) S3 Table. Within the per procedure costs, we also identified a percentage of spending specific to the RRI.

### Data analysis

We collected data on the total number of procedures completed from 2017–2018. Total number of procedures were grouped by age categories and by month of completion.

The focus of our analysis was on the unit cost of one facility-based procedure compared to an RRI-specific procedure. To estimate the average CPC conducted during routine care and the average CPC conducted within an RRI, we totaled all spending based on three categories: stable programmatic, RRI-specific, and per procedure costs. To account for spending that did not vary by procedure or if it occurred during an RRI, we calculated the total costs and divided it equally across the months within our study period. Next, we took the total spent on procedures-related expenses and divided them by the total number of VMMCs conducted over the year. We then allocated these expenses based on the number of VMMC conducted during each month of the study period.

In order to appropriately divide up the stable programmatic costs among the RRI and facility-based months, we accounted for 25% of the stable programmatic costs to be during RRI months and 75% to be during facility-based months since 3 of the 12 months were during RRI and the other 9 were facility-based and the costs should remain stable throughout. We accounted for 100% of the RRI-specific costs to occur during RRI months. Next, we calculated the procedures that occurred during the RRI months (December, April, and August) and non-RRI months. We then used the percentages of procedures to compare the per procedure costs during an RRI to facility-based VMMC.

### Sensitivity analysis

Although we expected high numbers of VMMC to occur during school holidays which are in December, April, August, there was an additional increase in VMMC conducted during other months. To address this overflow, we conducted a sensitivity analysis which assumed that RRIs occurred over 2 months instead of 1 to estimate the potential variation in the CPC for facility-based and RRI in this context. To do this, we totaled all spending that varied by procedure and divided it up for 6 RRI months (Nov, Dec, Mar, Apr, Jul, August) and the 6 non-RRI months (Oct, Jan, Feb, May, June, Sept).

## Results

**Fig 1:**
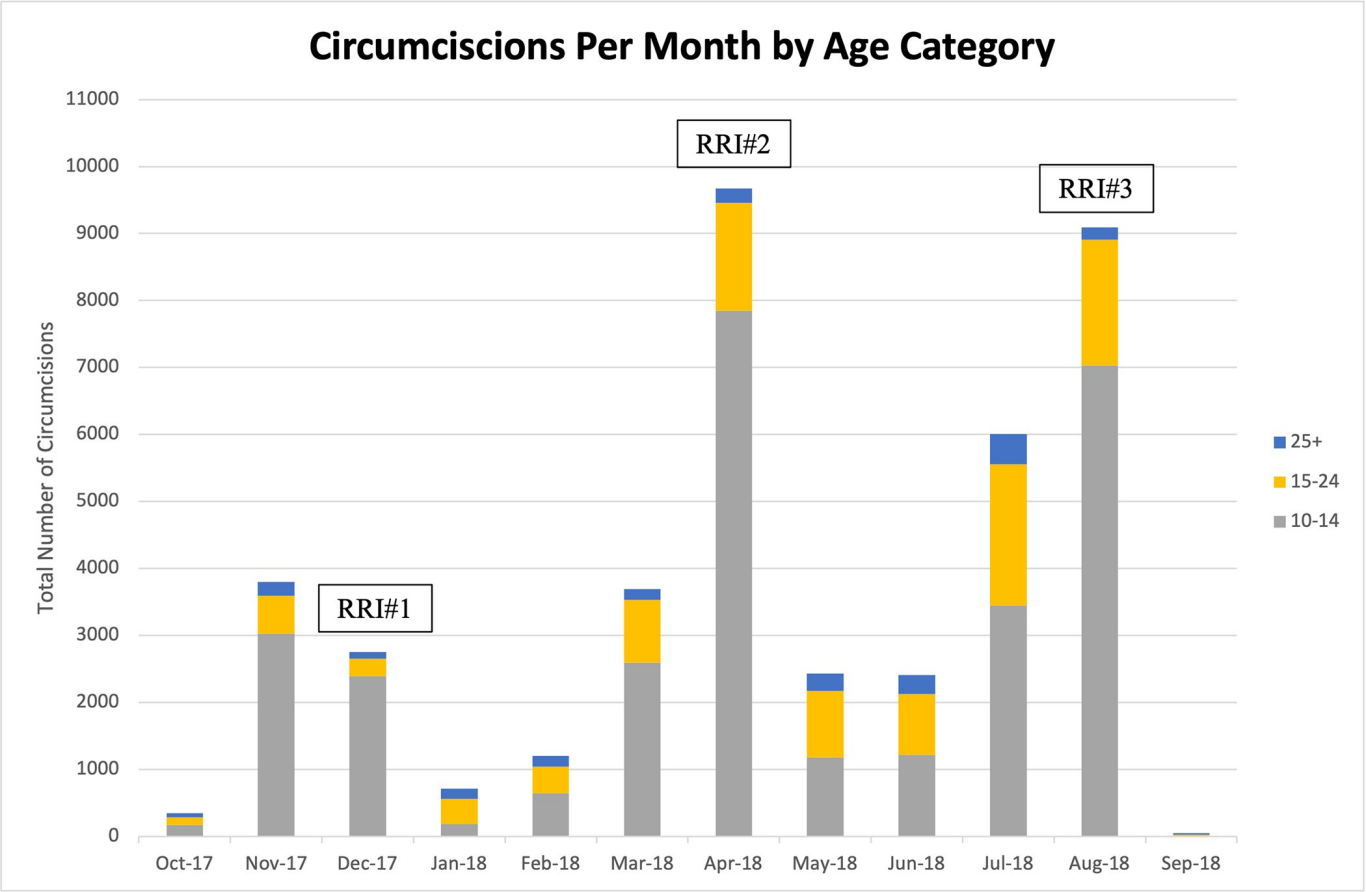
Circumcision per month by age category. Distribution of completed VMMC by month and age group (10–14, 15–24, 25+years old). Months specific to a Rapid Results Initiative are December 2017, April 2018, August 2018.

From October 2017-September 2018 staff conducted a total of 42,139 VMMCs. Of these, 21,514 (51%) were conducted during routine care (facility-based) and 20,625 (49%) were conducted during RRIs. The total number of VMMCs conducted and age distribution of clients circumcised varied by month. Some of the peaks occurred in December, April, and August coinciding with the school holidays and the months RRIs took place (Fig 1). Peaks in May and July coincided with half term breaks. Procedures for boys aged 10–14 years made up 70% (n = 29,712) of all procedures, while the older adolescents and young adults aged 15–24 years accounted for 24.1% (n = 10,175) of all procedures. Adults 25 years (n = 2252, 5.3%) of age and older accounted for the least number of procedures performed during this time period.

Overall, FACES spent $3,092,891 toward VMMC. This included $2,644,910 in stable programmatic costs, $308,195 in costs that were directly related to RRI activities (December 2017, April 2018, August 2018), and $139,786 in per procedure costs (Table 1).

**Table 1:** Spending per category.

	Facility-Based	RRI	Total
**Stable Programmatic Costs**			
Personnel	$1,054,974 (51.5%)	$351,658 (33.7%)	**$1,406,632**
Capital Goods	$42,545 (2.1%)	$14,181 (1.4%)	**$56,726**
Recurrent Goods	$198,794 (9.7%)	$66,265 (6.3%)	**$265,059**
Services	$631,708 (30.8%)	$210,569 (20.2%)	**$842,277**
Facility	$55,662 (2.7%)	$18,554 (1.8%)	**$74,216**
**RRI-specific costs**			
Personnel	$0 (0%)	$95,718 (9.2%)	**$95,718**
Capital Goods	$0 (0%)	$0 (0%)	**$0**
Recurrent Goods	$0 (0%)	$146,389 (14.0%)	**$146,389**
Services	$0 (0%)	$20,773 (2.0%)	**$20,773**
Facility	$0 (0%)	$45,315 (4.3%)	**$45,315**
**Per Procedure costs**			
Personnel	$0 (0%)	$0 (0%)	**$0**
Capital Goods	$0 (0%)	$0(0%)	**$0**
Recurrent Goods	$65,208 (3.2%)	$73,846 (7.1%)	**$139,055**
Services	$343 (0.02%)	$388 (0.04%)	**$731**
Facility	$0 (0%)	$0 (0%)	**$0**
**Total costs**			
Personnel	$1,054,974 (51.5%)	$447,376 (42.9%)	**$1,502,350**
Capital Goods	$42,545 (2.1%)	$14,181 (1.4%)	**$56,726**
Recurrent Goods	$264,003 (12.9%)	$286,500 (27.5%)	**$550,503**
Services	$632,051 (30.8%)	$231,730 (22.2%)	**$863,781**
Facility	$55,662 (2.7%)	$63,869 (6.1%)	**$119,531**
Stable Programmatic costs totals	$1,983,683 (96.8%)	$661,227 (63.4%)	**$2,644,910**
RRI-specific costs totals	$0 (0%)	$308,195 (29.5%)	**$308,195**
Per Procedure costs totals	$65,551 (3.2%)	$74,235 (7.1%)	**$139,786**
**Totals**	**$2,049,234**	**$1,043,657**	**$3,092,891**

Notes: Comparing Facility-Based to RRI by stable programmatic costs, RRI-specific costs, and Per Procedure Costs. The percentage represents the amount spent in the respective category (row) divided by the total spending for either facility-based or RRI (column).

Spending per month during an RRI varied with most of the spending allocated to personnel which accounted for 42.9% of spending and included per diem staff and consultants needed during the outreach (Table 1). The second highest spending category within RRI spending was for recurrent goods (27.5%) which included items such as office supplies, equipment, gas and oils, pharmaceuticals, etc. Per procedure costs for VMMC conducted during RRIs included $19.81 (42.9%) in personnel costs and $26.41 (57.1%) in all other costs compared to $51.68 (51.5%) in personnel costs and $48.70 (48.5%) all other costs for facility-based procedures. The unit cost per VMMC conducted during the months of an RRI was $48.51USD and the unit costs for months outside of RRI was $99.35 USD (Table 2). The CPC during an RRI is less than one that is facility-based. Cost analysis between age categories showed that CPC for 10-14- and 15-24-year old’s during an RRI was the least expensive, ($83.89 and $278.61, respectively; Table 3).

**Table 2:** Computation of the cost per circumcision by RRI and facility-based. Total spending by category was divided by the total completed procedures to result in the cost per circumcision.

	RRI	Facility-Based	Total
Total VMMC	21514	20625	42139
Total Costs	$1,043,657	$2,049,234	$3,092,891
CPC	$48.51	$99.36	$73.40

Note: VMMC, voluntary medical male circumcision; RRI, rapid results initiative; CPC, cost per circumcision.

**Table 3:** Computation of the cost per circumcision by age categories. Total number of procedures and total spending by each category (RRI vs facility-based) are broken down by age group. Cost per circumcision is then calculated by category for each age group.

Age Groups	Total VMMC	RRI VMMC	Facility Based VMMC	CPC RRI	CPC Facility	Difference in Spending
10–14	29712	17271	12441	$83.89	$164.72	(-) $80.83
15–24	10175	3746	6429	$278.61	$318.75	(-) $40.14
25+	2252	497	1755	$2,099.91	$1,167.65	$932.26

Note: VMMC, voluntary medical male circumcision; RRI, rapid results initiative; CPC, cost per circumcision.

### Sensitivity analysis

We then conducted a sensitivity analysis for CPC to account for deviation from the predicted values. The total number of VMMCs in November, December, March, April, June, and July were included. CPC for facility-based ranges from $99.35 to $287.25 and for RRI costs ranged from $29.81 to $48.51.

## Discussion

Our results show that the average cost of one VMMC in Kisumu County in Kenya during an RRI was $48.51 compared to $99.35 for a facility-based procedure. We also saw a high number of VMMC within childhood cases 10–14 years old in our RRI campaign. A study documenting the first RRIs conducted in former Nyanza province (which includes: Siaya, Kisumus, Homa Bay, Migori, Kisil, and Nyamira counties) in 2009 and 2010 reported a lower CPC than we observed, the demand was much higher at the time of rollout. RRI reported costs for VMMC within FACES fell below the range of recently published estimates [19, 20]. A study conducted in South Africa showed that the cost of VMMC during routine care was $130.10 and the cost of VMMC in a routine care facility with outreach services was $138.50 [23]. This lower cost within RRI could be due to efficiency, low overhead costs and increased volume compared to the facility-based model especially as it relates to school aged children. Focusing on RRI rather than spending more money on facility based VVMC remains an efficient method for implementation of VMMC.

Interestingly, 51% of the procedures were conducted during RRI, and children 10–14 years made up 70.5% of the total clients. Most of the procedures occurred during the school holiday months and RRIs; however, May and July also had high rates of VMMC due to half term breaks. Among regions/communities with high HIV prevalence that have reached saturation, focus might shift to boys within the 10–14-year-old cohort. Given that cost was less for males 10–24 years old during an RRI, this may serve as an important modality. Additionally, data has shown that targeting this age group is a good investment for HIV prevention prior to sexual activity [24, 25]. However, a recent reports showed that conventional circumcision presents an increased risk for glans injuries and urethral fistula in those younger than 15 years old, with a high incidence in those 6–14 years of age [20]. Kenya was one of the top three countries with fistulous injuries accounting for 5/41 total injuries reported between 2015–2019 [26]. Based on this recent finding, there has been a change in guidelines among VMMC efforts to focus on those 15 years and older. We must take the fact that these children live in high HIV prevalence areas as we weigh out the risks and benefits of offering this service to boys 10–14 years old. VMMC in this population is contingent upon implementation of strategies that minimize adverse events associated with VMMC which may include use of devices, post-surgical counseling, and emphasis on wound care [27].

Alternatively, despite the progress that Kenya has made with regards to VMMC, a gap still exists within Kenya in relation to older men, 15+, who represented 29.5% of total procedures. It is likely an integration model of RRI and facility-based outreach where cohorts of boys are all circumcised at the same time every one to three years as saturation is reached and newborns age into the VMMC eligible age category [28]. Another option would be to figure out a way to target older men in an RRI approach. One study reported that workplace initiatives in mining and commercial farming were put in place to increase reach among working men [29].

Although we observed substantial differences in the unit cost of VMMC, our estimates of the unit cost of both RRI and facility-based VMMC are limited. First, we were only able to track VMMC completion by month. Although the RRIs were focused in December, April, and August, increased VMMC also occurred during December, March, and July which coincided with other school holidays. Second, we were not able to track the exact date that specific resources were used. For example, certain payments were made in preparation for an RRI the month that preceded mobilization (e.g. site rental payments) while other costs were paid after completion of RRIs (e.g. staff per diems). We attempted to account for these differences by lagging VMMC by one month, however we could not account for all variability. Third, it is likely every individual did not have an equal chance of being a participant in our RRI intervention; younger males had more free time during an RRI as they were during school holidays compared to those who are older and more likely working.

## Conclusion

The highest number of VMMC were conducted during school holidays and term breaks. The majority of procedures during an RRI and term breaks were conducted in school aged children, 10–14 year old. Increasing capacity to conduct VMMC during these times of the year is an efficient approach to VMMC within this age group until implementation reaches scale. However, as we are conducting VMMC among emerging populations of adolescent and pre-adolescent males, it is imperative we figure out ways to continue engaging those older than 15 years old during holiday campaigns or through other outreach measures. While lower CPC does not ensure sustainability, scale up of RRI may be an efficient model for HIV prevention through circumcision in Kenya.

## Supporting information

S1 Table Spending items organized into one of the five spending categories as filed in the ledger. (PDF)

S2 Table Categorization of all spending into broad categories with a total of 49 different groups. (PDF)

S3 Table Spending organized into one of the three overall VMMC costing categories and items included in each category. (PDF)
