# Supplementary material for: Cost comparison of a rapid results initiative against standard clinic-based model to scale-up voluntary medical male circumcision in Kenya
Source: PLOS Glob Public Health. 2023 Mar 29;3(3):e0000817. doi: 10.1371/journal.pgph.0000817 (PMC10057778; doi:10.1371/journal.pgph.0000817)
Supplement: S1 Table — (PDF) [file pgph.0000817.s001.pdf]

S1 Table: Spending items organized into one of the five spending categories as filed in the ledger.

Spending Category	Items Included
**Recurrent Goods**	Gas and Oil<br>Software<br>Non-Medical Supplies<br>Insurance Expenses<br>Training Materials<br>Permits and Licenses<br>Office Supplies<br>Freight/Delivery/Installment/Postage<br>Research Supplies<br>Pharmaceuticals<br>Participant Incentives<br>Medical Supplies
**Capital Goods**	Furniture<br>Equipment<br>Computers
**Facility**	Venue Costs<br>Lodging<br>Rent
**Personnel**	Consultants<br>Salaries and Wages<br>Staff Per Diems<br>Independent Contractor<br>Mobilization - outreach worker<br>Fringe Benefits<br>Temporary Labor<br>Employer Contributions- global programs and adjusting journal entries (negative amount)<br>MOH<br>KEMRI
**Services**	Facility alterations<br>Other Research Cost<br>Printing and Reproduction<br>Internet, Data and Network<br>Transport Expenses<br>Travel, Non-Airfare<br>Utilities<br>Vehicle services- petty cash, car wash, motorcycle repair<br>Visa Fees<br>Workshop and Training<br>Airfare<br>Banking Fees<br>Cellular Communication

	Conference Registration<br>NITA<br>Repair Expenses<br>Sanitation<br>Security Services<br>VAT
