# Supplementary material for: Cost comparison of a rapid results initiative against standard clinic-based model to scale-up voluntary medical male circumcision in Kenya
Source: PLOS Glob Public Health. 2023 Mar 29;3(3):e0000817. doi: 10.1371/journal.pgph.0000817 (PMC10057778; doi:10.1371/journal.pgph.0000817)
Supplement: S2 Table — (PDF) [file pgph.0000817.s002.pdf]

S2 Table. Categorization of all spending into broad categories with a total of 49 different groups.

Categories				
Airfare	Facilities Management	KEMRI	Permits and Licenses	Training Materials
Banking Fees	Facility alterations	Lodging	Pharmaceuticals	Transport Expenses
Cellular Communication	Freight/Delivery/ Installment/Postage	Medical Supplies	Printing and Reproduction	Travel, Non-Airfare
Computers	Fringe Benefits	Mobilization	Rent	Utilities
Conference Registration	Furniture	MOH	Repair Expenses	VAT
Consultants	Gas and Oils	NITA	Research Supplies	Vehicle services
Employer Contributions	Independent Contractor	Non-Medical Supplies	Salaries and Wages	Venue Costs
Equipment	Insurance Expenses	Office Supplies	Sanitation	Visa Fees
Facilitation	Internet, Data and Network	Other Research Cost	Security Services	Workshop and Training
Staff Per Diems	Temporary labour	Participant Incentives	Software	

Notes: KEMRI, Kenya Medical Research Institute; MOH, Ministry of Health; NITA, National Industrial Training Authority; VAT, Value Added Tax.
