# Supplementary material for: Cost comparison of a rapid results initiative against standard clinic-based model to scale-up voluntary medical male circumcision in Kenya
Source: PLOS Glob Public Health. 2023 Mar 29;3(3):e0000817. doi: 10.1371/journal.pgph.0000817 (PMC10057778; doi:10.1371/journal.pgph.0000817)
Supplement: S3 Table — (PDF) [file pgph.0000817.s003.pdf]

S3 Table: Spending organized into one of the three overall VMMC costing categories and items included in each category.

VMMC Costing Category	Items Included
**Stable Programmatic Costs**	Research Supplies<br>Office Supplies<br>Freight/Delivery/Installment/Postage<br>Permits and Licenses<br>Training Materials<br>Insurance Expenses<br>Non-Medical Supplies<br>Software<br>Computers<br>Equipment<br>Furniture<br>Rent<br>Salaries and Wages<br>Fringe Benefits<br>KEMRI<br>MOH<br>Facility alterations<br>Other Research Cost<br>Printing and Reproduction<br>Transport Expenses<br>Utilities<br>Visa Fees<br>Workshop and Training<br>Airfare<br>Banking Fees<br>Cellular Communication<br>Conference Registration<br>NITA<br>Repair Expenses<br>Sanitation<br>Security Services<br>VAT<br>Internet, Data and Network
**RRI-Specific Costs**	Staff Per Diems<br>Mobilization/Outreach worker<br>Independent Contractor<br>Consultants<br>Temporary labor<br>Venue Costs<br>Lodging<br>Travel, Non-Airfare<br>Gas and Oils
**Per Procedure Costs**	Medical Supplies<br>Pharmaceuticals<br>Participant Incentives
